# Acute Effects of Transcutaneous Auricular Vagus Nerve Stimulation on Autonomic Nervous System Regulation and Spasticity in Children with Spastic Cerebral Palsy: A Preliminary Study

**DOI:** 10.3390/biomedicines14061370

**Published:** 2026-06-18

**Authors:** Gulay Yalcin, Gorkem Acar, Muhammed Fatih Kavak, Sevinç Külekçioğlu, Ali Veysel Özden

**Affiliations:** 1 Department of Physiotherapy and Rehabilitation, Mudanya University, 16940 Bursa, Turkey; 2 Faculty of Sport Science, Istanbul Gelisim University, 34310 Istanbul, Turkey; gacar@gelisim.edu.tr; 3 Department of Physiotherapy and Rehabilitation, Faculty of Health Sciences, Usküdar University, 34662 Istanbul, Turkey; fatih.kavak@uskudar.edu.tr; 4 VM Medical Park Hospital, 16220 Bursa, Turkey; sevinckulek@gmail.com; 5 Health Sciences Faculty, Bahcesehir University, 34353 Istanbul, Turkey; aliveysel.ozden@bau.edu.tr

**Keywords:** cerebral palsy, taVNS, spasticity, autonomic nervous system, heart rate variability

## Abstract

**Objective:** This study aims to investigate the acute effects of transcutaneous auricular vagus nerve stimulation (taVNS) on autonomic nervous system (ANS) regulation and spasticity in children with spastic cerebral palsy (SCP). **Methods:** This preliminary study includes 20 children aged 2–15 years diagnosed with SCP. Participants undergo a single session of taVNS. Spasticity is assessed using the Modified Ashworth Scale, and autonomic regulation is evaluated through heart rate variability (HRV) parameters measured before and immediately after stimulation. **Results:** Following taVNS, spasticity scores decrease significantly (Modified Ashworth Scale: pre 2.00 ± 0.64 vs. post 1.60 ± 0.52; *p* = 0.004). Significant reductions are also observed in mean heart rate (pre 98.60 ± 16.32 bpm vs. post 91.25 ± 20.22 bpm; *p* = 0.022), LF/HF ratio (pre 2.22 ± 2.22 vs. post 1.12 ± 0.84; *p* = 0.006), and LF power (*p* = 0.009). No significant changes are detected in RMSSD, pNN50, or HF power (all *p* > 0.05). No adverse events are reported. **Conclusions:** This preliminary study suggests that a single session of taVNS may be associated with acute changes in autonomic regulation and reductions in spasticity in children with SCP. The observed shifts in HRV parameters indicate a modulation of sympathovagal balance. These findings support the feasibility of taVNS as a non-invasive neuromodulatory approach and warrant further large-scale, controlled studies with longer follow-up. Because of the small sample size, the absence of a control or sham group, and the short (1-min) HRV recording window, these results should be regarded as preliminary and hypothesis-generating, and they require confirmation in larger, randomised, sham-controlled studies. Clinical Trial Registration: This study was registered at ClinicalTrials.gov (Identifier: NCT06880887) on 10 March 2025. The study was conducted between January 2025 and March 2025; registration on 10 March 2025 therefore occurred during the enrolment and data-collection period rather than prior to it. This retrospective registration is acknowledged as a limitation.

## 1. Introduction

Cerebral palsy (CP) is a common neurodevelopmental disorder that persists throughout life, resulting from permanent, non-progressive damage to the developing brain during the prenatal, perinatal, or postnatal period [1]. While the primary clinical feature of CP is motor dysfunction, a significant proportion of children also experience additional neurological and systemic complications such as epilepsy, sensory and cognitive problems, communication difficulties, and behavioural problems [1,2]. Recent studies have shown that CP affects not only the motor system but also has significant effects on the autonomic nervous system (ANS). In particular, it has been reported that sympathetic-vagal balance is disrupted in children with CP, which may pose additional risks in terms of cardiovascular morbidity and mortality [2,3].

Multiple mechanisms underlie ANS dysfunction in CP. These include the direct involvement of brain regions that play a regulatory role in cardiac functions (heart rate and vascular tone), the sedentary lifestyle associated with the physical activity limitations commonly observed in children with CP, and the accompanying cardiometabolic risk factors [3,4]. These findings demonstrate that focusing solely on motor rehabilitation is insufficient and that the cardiovascular health and quality of life of children with CP must also be included in a comprehensive assessment and intervention process.

Among neuromodulation approaches, vagus nerve stimulation (VNS) has emerged as one of the prominent methods in recent years. The vagus nerve exerts regulatory effects on the cholinergic and adrenergic systems via its afferent fibres, thereby supporting cortical plasticity and contributing to neurological recovery processes [5,6]. Clinical studies in individuals with stroke, traumatic brain injury, and spinal cord injury have shown that VNS, when applied in conjunction with rehabilitation, increases motor gains and supports cortical reorganisation [6,7,8,9].

However, studies examining VNS applications in children with CP are quite limited. The potential role in reducing spasticity, the effects on functional independence, and the lack of findings regarding ANS responses indicate a significant knowledge gap in this area [10]. Therefore, innovative treatment approaches targeting both the motor and autonomic nervous system components of CP are needed.

Therefore, this study aims to investigate the acute effects of transcutaneous auricular vagus nerve stimulation (taVNS) on autonomic nervous system regulation, assessed by heart rate variability parameters, and on spasticity in children with spastic cerebral palsy. This study is designed to provide preliminary evidence regarding the clinical applicability of taVNS in this population.

## 2. Material and Method

### 2.1. Research Design and Ethical Approval

This study was designed as a prospective single-group pretest-posttest clinical study. The research was conducted at the Physical Medicine and Rehabilitation Department of Bursa VM Medicalpark Hospital between January 2025 and March 2025. Approval for the study was obtained from the Istanbul Medipol University Non-Interventional Clinical Research Ethics Committee on 16 January 2025, under reference number E-10840098-202.3.02-526. All participants’ parents or legal guardians were informed about the research process and provided written consent.

### 2.2. Participants

Twenty children aged 2–15 years who had been diagnosed with spastic cerebral palsy by a physical medicine and rehabilitation specialist or neurologist were included in the study (Table 1). Participants were recruited consecutively from the outpatient clinic. Inclusion criteria for the study included having a diagnosis of spastic CP, being aged between 2 and 15 years, the child being willing to participate in the study, and the parent or legal guardian providing written consent, along with the absence of acute illness or comorbidity that could affect autonomic function or spasticity. Exclusion criteria included severe intellectual disability preventing understanding of the study procedures, presence of uncontrolled cardiovascular, respiratory, or autonomic disorders (e.g., arrhythmia or severe asthma), presence of medical devices that could be affected by VNS (e.g., pacemaker or cochlear implant), the risk of VNS interacting with antiepileptic drugs or triggering epileptic seizures, and non-compliance with the study protocol or lack of parent–child cooperation. In addition, the age, gender, height, weight, and body mass index of all participants were systematically recorded as sociodemographic characteristics. To minimise the influence of concomitant therapies on autonomic and spasticity outcomes, children who had received botulinum toxin injections within the previous three months, those whose oral antispastic medications (e.g., baclofen, tizanidine, diazepam) had been initiated or dose-adjusted within the previous month, and those who had started a new or intensified rehabilitation programme within the previous four weeks were excluded. All enrolled participants continued their ongoing routine physiotherapy schedule unchanged during the study period, and no participant received new pharmacological or rehabilitative interventions between the pre- and post-stimulation assessments.

### 2.3. Power Analysis

The sample size was calculated using G*Power software version 3.1.9.7 (Heinrich Heine University Düsseldorf, Düsseldorf, Germany). Considering the effect size d = 0.80, alpha = 0.05, and power (1 − β) = 0.80 parameters, the minimum sample size was determined to be 15. Therefore, the study was completed with 20 participants to prevent possible losses [11].

### 2.4. Measurement Tools and Data Collection Procedure

#### 2.4.1. Gross Motor Function Classification System (GMFCS)

Participants’ functional levels were assessed using the GMFCS. The GMFCS classifies children with CP into five levels based on their sitting and walking abilities: Level I (independent walking) to Level V (limited mobility despite assistive technology) [12].

#### 2.4.2. Autonomic Nervous System Activity

Autonomic nervous system activity was assessed non-invasively via the finger using the Elite HRV device (Elite HRV Inc., Asheville, NC, USA). During the measurement, children were tested for one minute in a sitting position with guided breathing. Elite HRV measures heart rate variability (HRV) and records parameters such as the root mean square of successive differences (RMSSD), the percentage of successive normal-to-normal intervals differing by more than 50 ms (PNN50), low-frequency power (LF Power), high-frequency power (HF Power), the low-frequency/high-frequency ratio (LF/HF ratio), and average heart rate [13,14]. Although the Task Force of the European Society of Cardiology recommends short-term recordings of approximately five minutes for the analysis of frequency-domain HRV, ultra-short-term recordings of one minute have been previously validated in paediatric and clinical populations for selected indices such as mean heart rate and RMSSD, and, to a more limited extent, LF power and the LF/HF ratio. A one-minute recording window was chosen here to match the operating protocol of the Elite HRV device and the tolerance and compliance limits of children with spastic cerebral palsy, who often have difficulty remaining still for longer periods. Frequency-domain indices derived from this short window (LF, HF, and LF/HF) should therefore be interpreted with caution, since ultra-short recordings reduce spectral resolution and may not fully capture the slower oscillations that contribute to LF power, potentially affecting the stability of LF and LF/HF estimates.

#### 2.4.3. Spasticity Measurement

Spasticity levels were assessed using the Modified Ashworth Scale (MAS). This scale scores from 0 (normal tone) to 4 (complete rigidity). Measurements were based on the resistance encountered during passive movement of the upper and lower extremities [15]. Specifically, MAS was applied to the elbow flexors, wrist flexors, knee extensors, and ankle plantar flexors bilaterally, yielding eight individual joint scores per participant. The reported composite MAS score represents the mean of all assessed joints. All assessments were performed by a single trained physiotherapist (G.Y.) who remained blinded to the sequence of pre- and post-intervention measurement forms to reduce rater bias. The distribution of pre-intervention MAS scores was as follows: score 1 (n = 2), score 1+ (n = 4), score 2 (n = 8), score 3 (n = 5), score 4 (n = 1). It should be noted that the MAS is an ordinal clinical scale with limited psychometric precision; its coarse gradations and susceptibility to inter-rater variability represent an inherent limitation of the present findings.

Primary outcomes were changes in HRV parameters and spasticity (MAS).

### 2.5. Intervention: Auricular Vagus Nerve Stimulation

**Figure 1:**
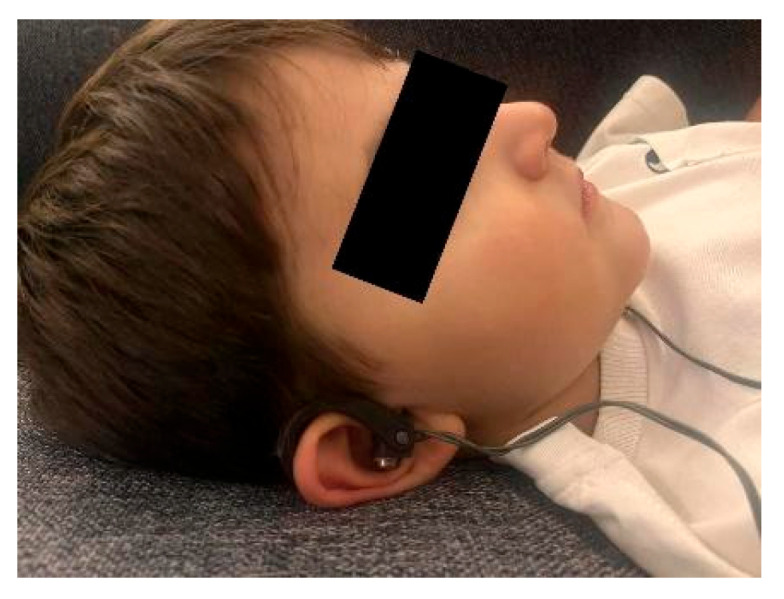
Representative application of bilateral transcutaneous auricular vagus nerve stimulation (taVNS) using the Vagustim device.

The intervention was delivered by a trained physiotherapist. Participants received a single session of transcutaneous auricular vagus nerve stimulation (taVNS), administered bilaterally using a Vagustim device (Vagustim Medical Technologies, Istanbul, Türkiye). Stimulation parameters were set at a frequency of 10 Hz, a pulse width of 300 microseconds, biphasic current, and a total duration of 20 min (Figure 1). Figure 1 displays a representative photograph of a child receiving the bilateral transcutaneous auricular vagus nerve stimulation (taVNS) using the Vagustim device, with electrodes positioned on the concha and tragus regions of both ears. Electrodes were placed on the concha and tragus regions, and stimulation intensity was gradually increased to the maximum level tolerated by each participant. The mean stimulation intensity applied was 0.8 ± 0.3 mA (range: 0.4–1.5 mA). All participants tolerated the procedure without premature discontinuation, and no adverse events were observed. In cases of discomfort or pain, the current intensity was immediately reduced to a comfortable level. All procedures were performed with participants in the supine position [16,17]. Blinding of participants and assessors was not applied due to the nature of the intervention and the pre–post study design. Based on the neuromodulatory role of the vagus nerve, it was hypothesized that taVNS would acutely improve autonomic regulation and reduce spasticity. Bilateral simultaneous stimulation was chosen for several reasons. First, the auricular branch of the vagus nerve (ABVN) on both sides projects to overlapping but partially distinct central pathways within the nucleus tractus solitarii, and bilateral activation is therefore expected to maximise afferent recruitment. Second, cardiac safety concerns (e.g., bradycardia) classically associated with selective right-sided cervical vagal stimulation are largely circumvented in transcutaneous auricular protocols, allowing safe bilateral application. Third, bilateral stimulation is the most frequently reported configuration in the existing taVNS literature on autonomic and motor outcomes, and was used here to enhance comparability with previous studies while acknowledging that laterality of stimulation may influence the magnitude and pattern of autonomic responses and warrants direct comparison in future trials [16,17].

### 2.6. Data Application and Collection Procedure

Ethical committee approval was obtained prior to the study, and written informed consent forms were obtained from the participants’ parents or legal guardians. After recording the participants’ sociodemographic information, such as age, gender, SP type, affected side, and educational status, their gross motor function levels were assessed using the GMFCS. The assessment sequence on the day of the intervention was as follows: (1) participants were seated for a 5-min rest period; (2) pre-intervention HRV was recorded in the sitting position (1 min); (3) pre-intervention MAS was assessed; (4) participants were repositioned to supine and taVNS was applied for 20 min; (5) immediately after stimulation ended, while still in the supine position, participants were assisted to the sitting position; (6) following a 5-min rest to allow postural stabilisation, post-intervention HRV was recorded in the sitting position; and (7) post-intervention MAS was subsequently assessed. Subsequently, taVNS was applied to all participants, and the same tests were repeated after the intervention to evaluate its acute effects.

### 2.7. Statistical Analysis

The data obtained from the study were transferred to IBM SPSS Statistics for Windows, Version 28.0 (IBM Corp., Armonk, NY, USA) and analysed. The distribution characteristics of the data were assessed using the Kolmogorov–Smirnov and Shapiro–Wilk tests. For variables demonstrating a normal distribution, differences between pre- and post-intervention measurements were evaluated using the paired samples *t*-test. For variables that did not follow a normal distribution, the non-parametric Wilcoxon signed-rank test was applied. Additionally, relationships between Gross Motor Function Classification System (GMFCS) levels and spasticity as well as autonomic nervous system parameters were examined using Spearman correlation analysis. All participants completed the study, and no missing data were observed. In all statistical analyses, a *p* value of <0.05 was considered statistically significant. Confidence intervals were not calculated, as the primary aim of this preliminary study was to explore acute within-subject changes. Effect sizes were calculated for all outcomes to supplement the *p*-value reporting: Cohen’s d (mean difference divided by the standard deviation of differences) was used for normally distributed paired comparisons, and the rank-biserial correlation r = Z/√N was used for Wilcoxon signed-rank tests. Effect sizes are reported alongside the test statistics in Table 2. Benchmarks of d or r ≥ 0.20, 0.50, and 0.80 were used to denote small, medium, and large effects, respectively. Given the exploratory and preliminary nature of this single-arm, hypothesis-generating study, no formal correction for multiple comparisons (e.g., Bonferroni adjustment or false discovery rate control) was applied to the analyses of HRV parameters and spasticity. Each outcome was treated as a pre-specified primary or secondary measure rather than as part of a confirmatory multiple-testing framework. Reported *p* values should therefore be interpreted descriptively, and the increased risk of type I error inflation is explicitly acknowledged as a limitation; the results require confirmation in adequately powered, controlled trials that incorporate appropriate corrections for multiplicity.

## 3. Results

All 20 participants completed the study, and no dropouts occurred. All participants were included in the analysis. Pre- and post-treatment measurements were compared (Table 2). A significant decrease in the Ashworth Scale score was observed after treatment (*p* = 0.004). A significant decrease in the average heart rate was also observed after treatment (*p* = 0.022). Significant decreases were found in LF power and the LF/HF ratio following treatment (*p* = 0.009 and *p* = 0.006, respectively). No significant differences were observed in HF power, RMSSD, or pNN50 values after treatment (*p* > 0.05). Effect sizes for the significant outcomes were large: Ashworth Scale (r = 0.64), LF Power (r = 0.58), and LF/HF Ratio (r = 0.62). For Average HR, a medium effect was observed (Cohen’s d = 0.56). Non-significant outcomes showed negligible effect sizes (RMSSD: d = 0.03; %PNN50: d = 0.01; HF Power: r = 0.01). Correlation analysis showed a strong and significant relationship between pre- and post-treatment Ashworth Scale scores (r = 0.684, *p* = 0.001). A strong and significant relationship was also observed between pre- and post-treatment average heart rate values (r = 0.760, *p* < 0.001). Correlation coefficients for LF power, HF power, and the LF/HF ratio were lower and showed varying levels of statistical significance. No adverse events or intervention-related complications were observed during or after the taVNS application.

The results of the correlation analysis between variables are shown in Table 3. A positive correlation was found between GMFCS and Ashworth Scale Difference (r = 0.246), but this relationship is not statistically significant (*p* = 0.296). A positive but low-level correlation was observed between GMFCS and Average HR Difference (r = 0.221, *p* = 0.349). A very weak negative relationship was detected between RMSSD Difference and GMFCS (r = −0.161), and this relationship was not statistically significant (*p* = 0.498). The correlation between the %PNN50 Difference and GMFCS was almost negligible (r = −0.007, *p* = 0.977). A low positive correlation was found between LF Power Difference and GMFCS, but this relationship was not statistically significant (r = 0.111, *p* = 0.642). A very weak negative correlation was found between HF Power Difference and GMFCS (r = −0.110, *p* = 0.645). Finally, the relationship between LF/HF Ratio Difference and GMFCS was positive but very weak (r = 0.179) and not statistically significant (*p* = 0.449). These results indicate that the effect of GMFCS on the variables examined is low and that no statistically significant correlation was found.

## 4. Discussion

Preclinical studies indicate that rapid vagus nerve stimulation (VNS), particularly at a frequency of 30 Hz and an intensity of 0.8 mA, applied following motor activity, accelerates the recovery of motor functions by enhancing cortical plasticity [4]. Studies in rat models have reported that VNS combined with sensory or motor training facilitates the reorganisation of cortical neurons and supports motor recovery [18]. In a study conducted in humans, VNS combined with intensive upper extremity rehabilitation was reported to provide greater improvement compared to rehabilitation alone and to lead to a significant increase in Fugl-Meyer Assessment-Upper Extremity scores [6]. Transcutaneous auricular vagus nerve stimulation (taVNS) increases parasympathetic activity by stimulating the vagus nerve branches in the auricle and can reduce the frequency and severity of epileptic seizures. The literature contains findings that taVNS is an effective method in the treatment of epilepsy and significantly reduces seizures [19].

The findings obtained in this study contribute significantly to the literature by demonstrating the acute effects of transcutaneous auricular VNS (taVNS) on autonomic nervous system parameters and spasticity in children with spastic cerebral palsy. The findings are particularly noteworthy for the significant improvements in heart rate variability (HRV) parameters and the trend towards reduced spasticity levels as assessed by the Ashworth Scale. These results suggest that taVNS may influence autonomic regulation and sympathovagal balance and may play a role in regulating muscle tone. Previous studies have similarly reported that taVNS modulates cardiac autonomic function and increases parasympathetic response [20,21]. Our findings are partially consistent with this literature. This interpretation must, however, be made with caution. Time-domain parasympathetic indices (RMSSD and pNN50) and HF power did not change significantly after the single taVNS session, so the observed effects appear to be driven primarily by the reduction in mean heart rate and the LF/HF ratio rather than by a uniform, robust enhancement of cardiac vagal modulation.

The findings regarding spasticity are particularly important from a clinical perspective. The fundamental mechanisms of spasticity in cerebral palsy include corticospinal tract damage, increased reflex activity, and decreased inhibitory mechanisms [22]. The literature suggests that taVNS may enhance spinal reflex inhibition via the locus coeruleus- noradrenergic system, thereby reducing muscle tone [23]. Significant reduction in spasticity observed in our study parallels results reported in both animal models and adult patient groups [17,18,19,20]. Furthermore, tVNS, used in the treatment of epilepsy and depression, has been reported to improve motor functions by increasing cortical plasticity [24]. These findings support the possible neurophysiological mechanisms underlying the reduction in spasticity scores observed in our study.

When HRV parameters were examined, a significant decrease in average heart rate was observed, which was interpreted as an indicator of increased vagal tone and parasympathetic activity. Similarly, the literature reports that taVNS increases parasympathetic dominance in heart rate regulation [25]. However, the lack of significant changes in time-based parasympathetic parameters such as RMSSD and pNN50 suggests that the acute effects of taVNS may manifest primarily through sympathovagal balance parameters (e.g., LF/HF ratio). Indeed, our study detected a significant decrease in the LF/HF ratio, which was interpreted as a suppression of sympathetic activity and a relative increase in parasympathetic activity. The significant decrease observed in LF power supports this mechanism, while the absence of a significant change in the HF parameter suggests that acute applications may only affect parasympathetic modulation to a limited extent [26]. Accordingly, statements regarding increased parasympathetic activity should be made with caution: because HF power, RMSSD, and pNN50 did not change significantly, the dominant acute effect of a single taVNS session in our sample is best characterised as a relative reduction in sympathetic outflow and a shift in sympathovagal balance, rather than as a clear-cut enhancement of cardiac vagal tone. Further studies with longer HRV recordings and repeated dosing are needed to determine whether more sustained or cumulative stimulation produces consistent changes in HF-related parasympathetic indices.

These findings are also consistent with the complex autonomic effects of vagus nerve stimulation. The observed decrease in average heart rate and improvement in the LF/HF ratio in our study may be explained by the simultaneous modulation of multiple autonomic pathways. Furthermore, it has been reported that taVNS may increase dopamine and noradrenaline release via catecholaminergic pathways, which may have positive effects on motor control and spasticity [27].

From a clinical perspective, current standard spasticity treatments (e.g., botulinum toxin, selective dorsal rhizotomy, physical therapy) generally provide only partial effects and are limited by side effects [27]. In contrast, taVNS stands out as an innovative alternative that can be integrated into multimodal rehabilitation protocols as a non-invasive, repeatable, and neuromodulatory method. The findings of our study suggest that taVNS may be a promising option for both spasticity management and autonomic nervous system regulation in children with cerebral palsy. However, the absence of significant changes in pure parasympathetic parameters such as RMSSD and HF suggests that the effect is primarily mediated through sympathovagal balance, particularly in the acute phase. Whether longer-term and repeated applications elicit different autonomic responses warrants investigation in future advanced studies.

The most important limitations of our study are the small sample size and the fact that only acute effects were evaluated. HRV and spasticity measurements were taken immediately after a single short-term application; thus, long-term effects could not be assessed. All participants tolerated the treatment well, and no side effects were observed. This design was planned primarily to examine the acute impact of taVNS in order to observe its tolerability and potential side effects. Moreover, treatment compliance can be challenging in pediatric populations, which may further affect the feasibility of long-term interventions. Several additional limitations should be acknowledged. First, the absence of a control or sham-stimulation group prevents firm causal attribution of the observed changes to taVNS itself; placebo, expectation, and habituation effects cannot be excluded. Second, the analysis was conducted without correction for multiple comparisons, which increases the risk of type I error and means that the reported *p* values should be interpreted descriptively. Third, the one-minute HRV recording window, although consistent with the device protocol and feasible in this paediatric population, is below the duration commonly recommended for reliable frequency-domain HRV analysis and limits the interpretability of LF power and the LF/HF ratio in particular. Fourth, although the eligibility criteria were designed to exclude children with recent botulinum toxin injections, recent changes in antispastic medication, or newly initiated rehabilitation programmes, residual confounding from ongoing routine physiotherapy and stable concomitant pharmacotherapy cannot be entirely excluded. Fifth, HRV measurements were obtained with participants seated, whereas the stimulation was delivered in the supine position. The postural transition between stimulation and post-assessment could have influenced HRV values independently of the intervention, since posture is a known determinant of autonomic tone; this represents a potential confound that should be controlled for in future studies by standardising the measurement posture throughout the protocol. Sixth, the trial was registered on 10 March 2025, during the enrolment and data-collection period (January–March 2025) rather than before the study commenced; this retrospective registration reduces confidence in the prospective nature of the outcome specification. Seventh, the MAS is a coarse ordinal scale with limited precision and susceptibility to inter-rater variability; future studies should incorporate more sensitive spasticity measures alongside or instead of the MAS. The present findings should therefore be regarded as preliminary and hypothesis-generating, and they require confirmation in adequately powered, randomised, sham-controlled trials that use longer HRV recordings, apply appropriate corrections for multiplicity, and explicitly standardise concomitant spasticity-related care.

## 5. Conclusions

This study contributes to the literature as one of the pioneering investigations examining the acute effects of taVNS on autonomic nervous system parameters and spasticity in children with spastic cerebral palsy. Our findings revealed that taVNS was associated with short-term changes in sympathovagal balance, reduced mean heart rate, and decreased spasticity levels assessed by the Ashworth Scale. In particular, the significant decrease in the LF/HF ratio and the drop in LF power values may indicate relative modulation of sympathovagal balance. These conclusions are preliminary and hypothesis-generating. Given the small sample, the single-arm uncontrolled design, the absence of a sham comparator, and the short HRV recording window, the magnitude, durability, and clinical relevance of these effects require confirmation in larger, randomised, sham-controlled studies.

These results suggest that taVNS may be a potential complementary treatment method for children with cerebral palsy, not only for autonomic nervous system regulation but also for spasticity control. With its non-invasive, safe, and repeatable characteristics compared to current invasive and pharmacological approaches, taVNS is considered an innovative option that can be integrated into multimodal rehabilitation protocols.

However, our study only addressed acute effects, and the potential contributions of long-term applications to spasticity and autonomic regulation should be tested in future randomised controlled trials. Large-scale, long-term studies will provide stronger evidence regarding whether taVNS can be routinely used in clinical practice.

## Figures and Tables

**Table 1:** Sociodemographic Characteristics of Participants.

Variable	N (%)	Mean ± SD	Min	Maximum
**Gender** **Female**				
	8(40)			
**Male**	12 (60)			
**Total**	20 (100)			
**Age (years)**	20 (100)	8.25 ± 3.97	2	15
**Height (cm)**	20 (100)	128.45 ± 23.80	75	160
**Weight (kg)**	20 (100)	27.90 ± 14.07	8	60
**Body Mass Index**	20 (100)	17.88 ± 6.68	8.63	35.28
**GMFCS:**		Median: 1 (IQR: I–III)	1	4
**I**	14 (70)			
**II**	1 (5)			
**III**	2 (10)			
**IV**	3 (15)			
**V**	0 (0)			

GMFCS: Gross Motor Function Classification System; N: Number of cases; SD: Standard deviation. Continuous variables are reported as Mean ± SD. GMFCS level, being an ordinal variable, is reported as Median (IQR) rather than Mean ± SD.

**Table 2:** Comparison of Pre- and Post-Treatment Values and Correlation Analysis Results.

Variables	Before (Mean ± SD)	After (Mean ± SD)	Difference (Mean ± SD)	t/Z Value	*p* Value
**Ashworth Scale**	2.00 ± 0.649	1.60 ± 0.528	0.40 ± 0.320	Z = −2.859 ^‡^	0.004 *
**Average HR**	98.60 ± 16.32	91.25 ± 20.22	7.35 ± 13.16	t = 2.496	0.022 *
**RMSSD**	86.23 ± 35.97	85.06 ± 36.44	1.17 ± 47.49	t = 0.110	0.913 ^†^
**%PNN50**	42.50 ± 17.72	42.15 ± 17.77	0.35 ± 24.91	t = 0.063	0.951 ^†^
**LF POWER**	14,982.38 ± 31,306.10	3574.24 ± 3517.93	11,408.14 ± 6954.50	Z = −2.613 ^‡^	0.009 *
**HF POWER**	8953.97 ± 21,443.40	4011.02 ± 4540.23	4942.97 ± 17,782.00	Z = −0.037 ^‡^	0.970
**LF/HF RATIO**	2.22 ± 2.22	1.12 ± 0.84	1.10 ± 1.18	Z = −2.763 ^‡^	0.006 *

HR: Heart Rate; RMSSD: Root Mean Square of Successive Differences; PNN50: Percentage of successive NN intervals differing by more than 50 ms; LF: Low Frequency; HF: High Frequency; * *p* ≤ 0.05; ^†^: Paired *t*-test; ^‡^: Wilcoxon Test. Effect sizes: Cohen’s d for paired *t*-test variables; rank-biserial r (=Z/√N) for Wilcoxon variables. Ashworth Scale: r = 0.64 (large); Average HR: d = 0.56 (medium); RMSSD: d = 0.03 (negligible); %PNN50: d = 0.01 (negligible); LF Power: r = 0.58 (large); HF Power: r = 0.01 (negligible); LF/HF Ratio: r = 0.62 (large).

**Table 3:** Correlation Analysis Results Between Variables.

Variables	GMFCS	Ashworth Scale Difference	Average HR Difference	RMSSD Difference	%PNN50 Difference	LF Power Difference	HF Power Difference	LF/HF Ratio Difference
**GMFCS**	1.000	0.246	0.221	−0.161	−0.007	0.111	−0.110	0.179
**Ashworth Scale Difference**	0.246	1.000	0.136	0.008	0.223	0.265	0.148	−0.114
**Average HR Difference**	0.221	0.136	1.000	0.099	0.185	0.230	0.300	−0.165
**RMSSD Difference**	−0.161	0.008	0.099	1.000	0.829 **	0.639 **	0.650 **	−0.370
**%PNN50 Difference**	−0.007	0.223	0.185	0.829 **	1.000	0.638 **	0.749 **	−0.390
**LF Power Difference**	0.111	0.265	0.230	0.639 **	0.638 **	1.000	0.513 *	−0.021
**HF Power Difference**	−0.110	0.148	0.300	0.650 **	0.749 **	0.513 *	1.000	−0.528 *
**LF/HF Ratio Difference**	0.179	−0.114	−0.165	−0.370	−0.390	−0.021	−0.528 *	1.000

HR: Heart Rate; RMSSD: Root Mean Square of Successive Differences; PNN50: Percentage of successive NN intervals that differ by more than 50 ms; LF: Low Frequency; HF: High Frequency; Spearman Correlation Coefficient; GMFCS: Gross Motor Function Classification System * *p* < 0.05; ** *p* < 0.01.

## Data Availability

The datasets generated and analyzed during this study are available from the corresponding author (Gülay Yalçın, gulay.yalcin@mudanya.edu.tr) upon reasonable request, subject to approval by the Mudanya University IRB to ensure compliance with data protection regulations. The raw data supporting the findings are partially included in the manuscript (e.g., summary tables) but are not publicly archived due to ethical restrictions on participant privacy.
